# Meloxicam inhibits biofilm formation and enhances antimicrobial agents efficacy by *Pseudomonas aeruginosa*

**DOI:** 10.1002/mbo3.545

**Published:** 2017-11-27

**Authors:** Pengfei She, Yangxia Wang, Zhen Luo, Lihua Chen, Ruichen Tan, Yanle Wang, Yong Wu

**Affiliations:** ^1^ Department of Clinical Laboratory The Third Xiangya Hospital of Central South University Changsha China; ^2^ Department of Clinical Laboratory The First Affiliated Hospital of Zhengzhou University Zhengzhou China

**Keywords:** biofilm, meloxicam, *Pseudomonas aeruginosa*, quorum sensing, synergistic effect

## Abstract

Microbial biofilms are communities of surface‐adhered cells enclosed in a matrix of extracellular polymeric substances. Bacterial cells in biofilm are 10~1,000‐fold more resistant to antimicrobials than the planktonic cells. Burgeoning antibiotic resistance in *Pseudomonas aeruginosa* biofilm has necessitated the development of antimicrobial agents. Here, we have investigated the antibiofilm effect of meloxicam against *P. aeruginosa* PAO1 and its potential mechanisms. Further, we have explored whether meloxicam could enhance the susceptibility of bacterial biofilms to treatment with conventional antimicrobials. Here, we found that meloxicam could significantly inhibit PAO1 biofilm formation in a dose‐dependent manner at the concentration without influence on planktonic cell growth. Meloxicam could also significantly inhibit the motilities, production of extracellular matrix, and expression of quorum sensing‐related genes and virulence factors of PAO1. Furthermore, synergistic interaction was observed when meloxicam combined with tetracycline, gentamicin, tobramycin, ciprofloxacin, ceftriaxone, ofloxacin, norfloxacin, ceftazidime, and DNase at subminimal inhibitory concentrations against PAO1 bioiflm. Collectively, our study lays the foundation for further investigation of repurposing meloxicam as a topical antibiofilm agent to treat *P. aeruginosa* biofilm‐related infections.

## 1 INTRODUCTION

Biofilm is a community of bacteria that are attached to a substratum or surface (Chen, Yu, & Sun, 2013)**,** typically consist of densely packed, multispecies populations of cells, encased in a self‐synthesized polymeric matrix, such as exopolysaccharides (EPS), extracellular protein, and extracellular DNA (eDNA), and attached to a tissue or surface (Costerton et al., 1987; Stoodley, Cargo, Rupp, Wilson, & Klapper, 2002). Cells embedded in biofilm are up to 1,000 times more resistant to conventional antibiotics compared to their counterparts (Mah & O'Toole, 2001). Biofilms have also been implicated as being involved in many different chronic bacterial infections, such as cystic fibrosis and chronic wound infection. Moreover, the bacteria could also cause infections in indwelling medical devices, such as catheters and artificial joint implants (Høiby et al., 2015).

*Pseudomonas aeruginosa* has become one of the most important model organisms for the study of biofilms due to the relative ease of growing consistent and reproducible structured biofilms under laboratory conditions, as well as its relevance as an opportunistic pathogen in hospital settings (Rybtke, Hultqvist, Givskov, & Tolker‐Nielsen, 2015). *Pseudomonas aeruginosa* biofilm formation could be largely influenced by quorum sensing (QS) system, which is a mechanism that bacteria use to coordinate their behavior. The *las* and *rhl* QS system is mediated by acyl‐homoserine lactone (HSL) signals including C4‐HSL and 3‐oxo‐C12‐HSL, which could activate the transcriptional regulators LasR and RhlR, respectively. The genes regulated by LasR and RhlR could control the biofilm formation, motility phenotype, and other virulence genes. Besides, recently studies have found that the compound 2‐heptyl‐3‐hydroxy‐4‐quinolone (*Pseudomonas* quinolone signal [PQS]) is a third QS signal. PQS was found to regulate the biofilm formation and the antioxidative properties of *P. aeruginosa* by serving as a link between the *las* and *rhl* QS system. Besides, QS also plays a pivotal role in antibiotic resistance and virulence (Balasubramanian et al., 2011; Lidor, AI‐Quntar, Pesci, & Steinberg, 2015).

Conventional antimicrobials have limited effectiveness against biofilm‐related infections, which increase the emergence of multidrug‐resistant *P. aeruginosa* (Walters, Roe, Bugnicourt, Franklin, & Stewart, 2003). Thus, the discovery of new anti‐infective agents, actively against microorganisms embedded in biofilms is an important goal in the treatment of biofilm‐related infection (Projan & Youngman, 2002). Interestingly, several studies have shown that certain nonsteroidal anti‐inflammatory durgs (NASIDs) possess antimicrobial activity. Chen and Wen (2011) found that treatments including administration of antibiotics together with NASIDs are active against bacteria in the biofilm. It was demonstrated that NASIDs, such as acetylsalicylic acid or diclofenac, characterize with bactericidal reaction on bacteria in the planktonic cells as well as biofilm (Dutta et al., 2008; Reslinski, Dabrowiecki, & Glowacka, 2015; Wang et al., 2003).

To the best of our knowledge, there is only one publication claimed that meloxicam (Melo) can interact with active sites of PQS system by a molecular docking assay (Soheili, Bazzaz, Abdollahpour, & Hadizadeh, 2015), but no other experimental data was found to prove the antibiofilm effect of Melo on *P. aeruginosa*. In this study, we evaluated the effect of Melo against *P. aeruginosa* biofilm formation and its mechanism in vitro. Further, we investigated the synergistic antibiofilm effect of Melo combined with conventional antibiotics (tetracycline, TE; gentamycin, GEN; tobramycin, TOB; ciprofloxacin, CIP; ceftriaxone, CTRX; ofloxacin, OFLX; norfloxacin, NFLX; and ceftazidime, CAZ) and DNase on biofilm formation.

## 2 MATERIALS AND METHODS

### 2.1 Bacterial strain and growth condition

PAO1 was used in the biofilm formation assay and was cultured in Luria–Bertani (LB) medium (Luqiao, Beijing, China). Melo was purchased from Aladdin (Shanghai, China), and dissolved in dimethyl sulfoxide (DMSO); In order to eliminate the effect of organic solvent on the results of our study, LB containing 1% DMSO was set as a negative control (NC); TE, TOB, CTRX, OFLX, NFLX, CAZ, and DNase were purchased from Aladdin (Shanghai, China), and dissolved in distilled water.

### 2.2 Effects of Melo on biofilm formation

The inhibitory effects of Melo on biofilm formation of *P.  aeruginosa* were evaluated by a semiquantitative plate assay as described earlier, with minor modifications (Liu et al., 2015; Nesse, Berg, & Vestby, 2015). Briefly, 4 μl overnight culture was added to 196 μl LB with the Melo at the designed concentration to be tested, and LB containing 1% DMSO was set as NC. After static incubation at 37°C for 24 hr, the plates were washed gently three times with saline to remove unattached bacteria, and stained with 0.5% (w/v) crystal violet (CV) for 15 min at room temperature. Then, the plates were washed again to remove the unbonded CV, 200 μl 95% ethanol was added to dissolve the CV, and the A570 nm of the wells was determined using a 96‐well plate spectrophotometer (Bio‐Tek, USA).

### 2.3 Evaluation of growth curve

The planktonic cell growth assay was modified from (Huang et al. (2014) as described previously. Overnight‐grown *P. aeruginosa* cells (adjusted to 0.5 McF) were diluted 1:100 in LB with Melo at the designed concentration to be tested. Triplicate samples were grown in sterile 50 ml centrifugal tubes (Corning/Costar, USA) at 37°C with agitation (160 rpm), and 200 μl culture medium was carefully transferred to a microplate every 4 hr for 20 hr in total. The planktonic culture turbidity was read at 630 nm (A630 nm) by the spectrophotometer.

### 2.4 Visualization of 24 hr biofilms

For visualization of biofilms, the procedure is briefly described as follows: Overnight‐grown *P. aeruginosa* cells were diluted 1:100 in Melo at the designed concentration to be tested in six‐well cell culture plates (Corning/Costar, USA) with cover slides, these plates were then statically incubated at 37°C for 24 hr to form biofilms. After incubation, the culture medium was removed and the cover slides were washed with saline for three times, then the cover slides were stained with CV as described above. The biofilm was visualized and photographed with an optical microscope (Olympus CX31, Tokyo, Japan) at the magnification of 400 ×.

### 2.5 Motility assays

Swimming, swarming, and twitching motility were assessed as previously described (Rashid & Kornberg, 2000). To investigate swarming motility, nutrient broth with 0.5% (wt/vol) Difco Bacto agar and different concentrations of Melo was used. A quantity of 4 μl of overnight cultures of *P. aeruginosa* grown in LB (1 × 10^9^ cells) was placed in the center of agar. After incubation for 24 hr at 37°C, the swarming zone diameter was measured. Swimming motility was evaluated using tryptone broth contained 0.3% (wt/vol) agarose. Swim plates were inoculated with *P. aeruginosa* grown overnight in LB agar (1.5%, wt/vol) at 37°C with a sterile toothpick. After incubation for 14 hr at 30°C, the swimming zone diameter was measured. For twitching motility, LB broth containing 1.5% agar was inoculated with a toothpick by stabbing the plates. After incubation at 37°C for 24 hr, the LB agar media was removed from the plates and then stained with 0.5% CV for 20 min. Spreading of *P. aeruginosa* from the inoculation point and the length of the “dendrite” were observed by naked eye and microscope (400 × ), respectively.

### 2.6 Colony morphology assay

A colony morphology assay was performed as the method described earlier, with minor modifications (Kim & Park, 2013). Briefly, 5 μl overnight culture of PAO1 in LB broth with (15.63 or 31.25 μg/ml) Melo or with 1% DMSO was added onto Congo red plates (1.5% agar, 10 g/L tryptone, 20 μg/ml Coomassie brilliant blue, and 40 μg/ml Congo red) and incubated at 25°C statically for 4 days.

### 2.7 Evaluation eDNA

The semiquantification of eDNA in the PAO1 biofilm was assessed by agarose gel electrophoresis (Gnanadhas, Elango, Datey, & Chakravortty, 2015). Briefly, treated or untreated 24‐hr‐old PAO1 biofilm was scraped from the six‐well cell culture plates, and sonicated for 15 min to release the eDNA combined to PAO1 biofilm. Then, the mixture was centrifuged at 2,000*g* for 5 min and the supernatant was loaded in the agarose gel (1%) and visualized, and grayscale value was calculated by ImageJ.

### 2.8 Detection of QS‐related virulence factors

Experiment was conducted based on our previous study (Qu et al., 2016). Briefly, overnight culture of PAO1 was inoculated into LB broth with (15.63 μg/ml) and without Melo and incubated for 16 hr, and then centrifuged at 4,000*g* for 15 min and the supernatant was filter sterilized (0.22 μm filter). Chloroform (in the ratio of 2:3) was used to extract pyocyanin pigment, and the pyocyanin pigment was reextracted with 1.0 ml of 0.2 mol/L HCl and absorbance was read at 540 nm (A540 nm). For elastase activity determination, 750 μl supernatant was incubated with 250 μl elastin Congo red solution (5 mg/mL, pH = 8.0) for 16 hr. Then, the mixture was centrifuged at 3,000 g for 10 min to remove unreacted precipitate, and debris and the absorbance was read at 490 nm (A490 nm). For protease activity detection, culture supernatant and 2% azocasein solution (pH = 7.0) were incubated in the ratio of 1:1 for 1 hr. A quantity of 500 μl 10% trichloroacetic acid was added to stop the reaction. Then, the mixture was centrifuged at 8,000*g* for 5 min and the absorbance of supernatant was read at 405 nm (A405 nm) (Kim & Park, 2013).

### 2.9 RNA extraction, cDNA synthesis, and comparative qPCR

The gene expression analysis was performed according to our previous study (Qu et al., 2016). Briefly, overnight cultures were grown to an A630 nm of 0.5–0.8 (37°C, 150 rpm) in LB broth with (15.63 μg/ml) or without (but 2% DMSO was added) Melo. And the total RNA was extracted using a E.Z.N.A. Total RNA Kit II (Omega Bio‐tek, Norcross, GA). RNA purity and concentration were determined by the absorbance at 260 and 280 nm, and 1 μl RNA was used for cDNA synthesis by the TransScript All‐in‐One First‐Strand cDNA Synthesis SuperMix for qPCR (Transgene, Beijing, China). A qPCR was performed with TransStartTM Green qPCR SuperMix UDG (Transgene, Beijing, China) using a real‐time quantitative PCR system (Eppendorf, Germany). The oligonucleotide primers used to amplify the QS‐related genes (*lasR*,* rhlR*,* mvfR*, and *pqsC/D*) and EPS‐related genes (*pslA*,* pelA*, and *alg44*) were referred to Qu et al. (2016) and Kim, Park, and Lee (2015), respectively.

### 2.10 Minimum Inhibitory Concentration (MIC)

The MIC was determined by the broth microdilution method as previously described by The Clinical and Laboratory Standards Institute (CLSI 2006). Concentrations for PAO1 ranged from 1,000 to 0.012 μg/ml for antimicrobial standards. Twofold dilutions were obtained in 96‐well plates with 100 μl of Mueller Hinton II (MH) broth per well. Then, the bacterial suspension (10 μl) was inoculated, and the plates were incubated for 16~20 hr at 35°C. The lowest concentration with any visible bacterial growth was taken as the MIC.

### 2.11 Synergistic biofilm inhibitory assay

Interference of biofilm formation upon treatment with Melo alone or in combination with antimicrobials was performed as the method described earlier, with minor modifications (Das et al., 2016). Briefly, 4 μl overnight‐grown *P. aeruginosa* cells was directly added to 196 μl LB containing subminimum inhibitory concentration (sub‐MIC) dose of TE (3.125 μg/ml), TOB (0.024 μg/ml), CTRX (3.125 μg/ml), OFLX (3.125 μg/ml), CAZ (0.781 μg/ml), NFLX (0.781 μg/ml), and DNase (6.250 μg/ml) alone and in combination with Melo (15.63 or 31.25 μg/ml) and incubated at 37°C for 24 hr. All test plates were washed, stained with CV as described above, and then the absorbance at 570 nm was measured.

### 2.12 Statistical analysis

Differences between groups were tested using a two‐tailed, unpaired Student's *t* test or one‐way ANOVA using GraphPad Prism 6.0 software. Differences were considered statistically significant when *p < *.05. All experiments were performed in triplicate and repeated three times.

## 3 RESULTS

### 3.1 Antibiofilm activity of Melo

**Figure 1:**
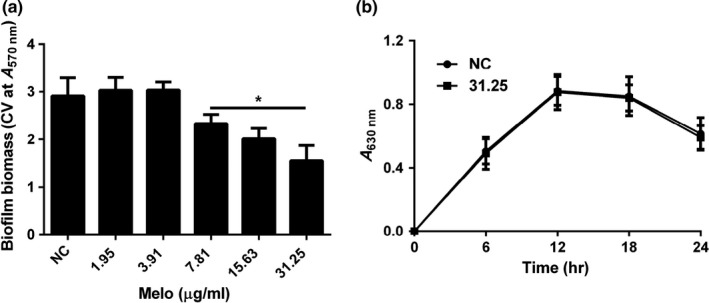
Effect of Melo on biofilm and planktonic cell of PAO1. (a) Biofilm formation was indicated by A570 nm in microplate with CV staining; (b) Growth curves of PAO1 with and without Melo. Total planktonic cell growth was traced by measuring A630 nm. Bars indicated standard error (SE) of the mean. * indicates that the mean A570 nm is statistically different from the control group with *p *<* *.05

**Figure 2:**
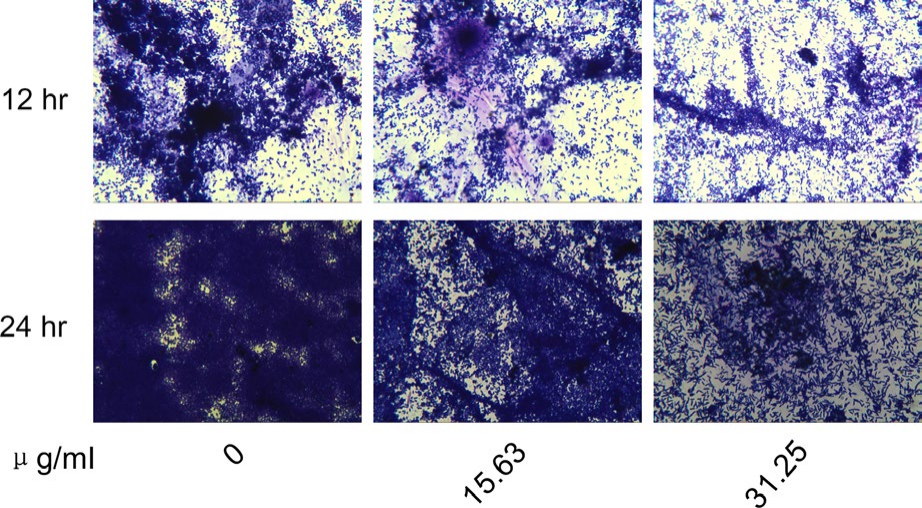
Visualization of biofilm. PAO1 biofilm was grown with various concentration of Melo on cover slides for 12 and 24 hr, respectively, and then stained with CV and observed by optical microscope (400 ×)

CV staining assays revealed that Melo could significantly inhibited PAO1 biofilm formation at the concentration of 7.81 μg/ml (*p *<* *.05) (Figure 1a) in a dose‐dependent manner but Melo did not affect planktonic cell growth at the concentration of ≤31.25 μg/ml (Figure 1b). To exclude diminutions of biofilm formation due to inhibitory effects on bacterial growth, sub‐MIC (≤31.25 μg/ml) of Melo was selected for the rest of the experiment. Microscopic images of biofilm formed by PAO1 in the presence of different concentrations of Melo (0~31.25 μg/ml) and stained with CV showed that the biofilm formation was inhibited in a dose‐dependent manner and the bacterial cells scattered singly on the adherent surface (Figure 2). Both the semiquantitative assays and microscopic images demonstrated that Melo could prevent *P. aeruginosa* biofilm formation.

### 3.2 Melo inhibits PAO1 motilities

**Figure 3:**
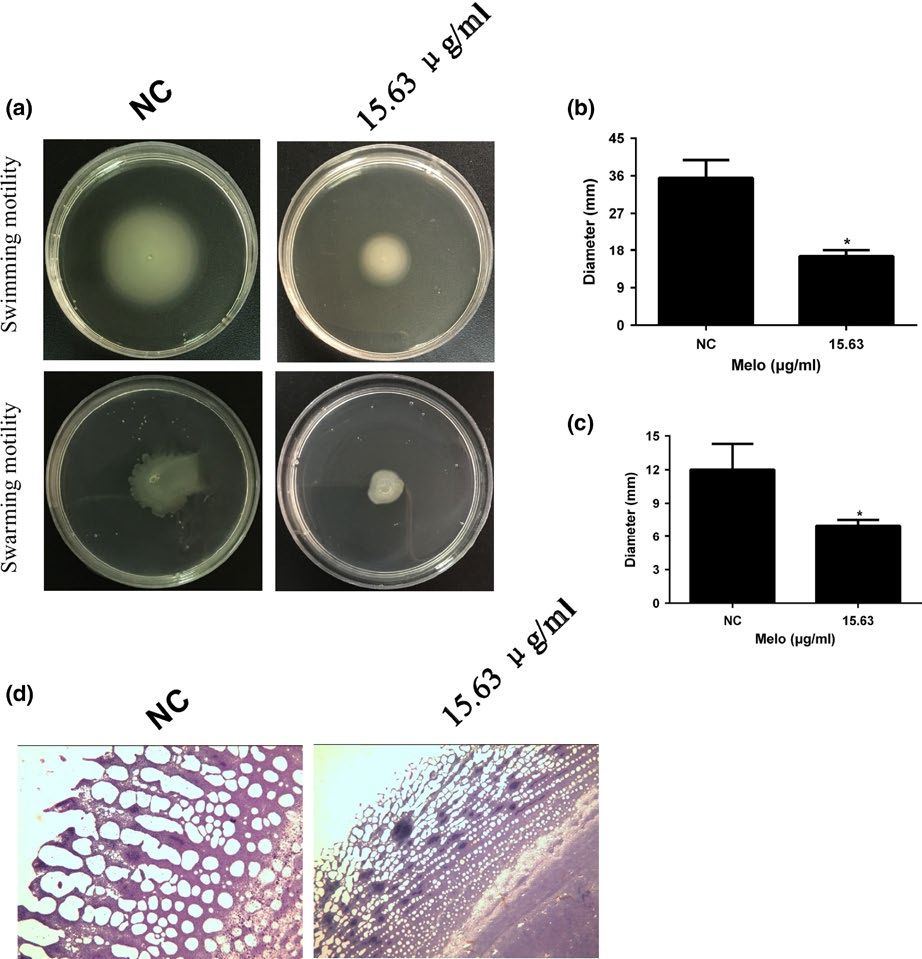
Inhibitory effect of Melo on motilities of PAO1. (a) Effects of Melo on swimming and swarming motility of PAO1 at the concentration of 31.25 μg/mL. Diameters of swimming (b) and swarming (c) zones were determined. And the phenotype of the “twitching antenna” was observed by microscope (400 ×) (d). Bars indicated SE of the mean. * indicates that the mean A570 nm is statistically different from the control group with *p *<* *.05

PAO1 displays several forms of motility on wet agarose including swimming, swarming, and twitching motility. The motilities of PAO1 cells grown with (31.25 μg/ml) and without Melo were evaluated by growing cells on several motility plates and by measuring the length of diameters (Figure 3a–c). Swimming motility zone of PAO1 without supplementation of Melo was 35.50 ± 4.30 mm that was significantly reduced to 17.00 ± 2.10 (*p* < .05) mm in the presence of 31.25 μg/ml Melo (Figure 3a and b); Similarly, the swarming zone was reduced from 12.00 ± 2.31 mm to 6.90 ± 0.71 mm (*p* < .05) in the presence of 31.25 μg/ml Melo (Figure 3a and c). Although, there was not a slight reduction of twitching zones observed by CV staining from naked eye (data not shown), but the length of the dendrites formed by PAO1 on twitching plates was significantly decreased in the presence of 31.25 μg/ml Melo observed by a microscope (Figure 3d).

### 3.3 Effect of Melo on extracellular matrix of PAO1 biofilm

**Figure 4:**
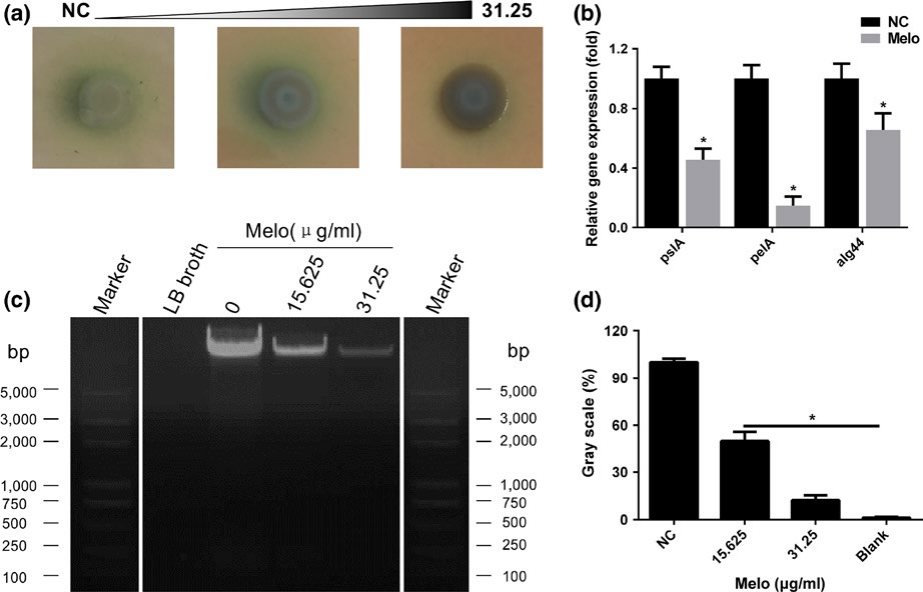
Effect of Melo on extracellular matrix of PAO1 biofilm. (a) Comparison of colony morphology on Congo red agar plates with different concentrations of Melo. (b) Melo decreased EPS‐related genes (*pslA, pelA*, and *alg44*) expression at the concentration of 15.63 μg/mL. (c) Effect of Melo on eDNA production determined by agarose gel electrophoresis (the white lines indicated the blank bands were removed, and the left reference band was a copy of the right reference band) and analyzed by the software of ImageJ (d). Bars indicated SE of the mean. *indicates that the mean A570 nm is statistically different from the control group with *p *<* *.05

Colony morphology was observed on an agar plate containing Congo red (Figure 4a). The colony grown on the plate without meloxicam showed a rugose morphology with reduction of pigment around the periphery known to be related to EPS overproduction. However, the colony became moist and smoother in the presence of 15.62 and 31.25 μg/ml Melo. At the same time, qPCR analysis for EPS‐related genes showed that Melo (15.62 μg/ml) could significantly inhibit the genes expression of *pslA* (*p* < .05), *pelA* (*p* < .05), and *alg44* (*p* < .05) (Figure 4b). Besides, earlier reports have shown that *P. aeruginosa* biofilm has eDNA as one of the major component (Flemming & Wingender, 2010). In our study, 24‐hr‐old biofilm culture supernatant was loaded in a 1% agarose gel and it was visible that Melo treated (15.62 and 31.25 μg/ml) biofilm culture supernatant possessed less eDNA than untreated group in a dose‐dependent manner(Figure 4c). The gray scale analysis of the bands by ImageJ was consistent with the electrophoresis results(Figure 4d). These results suggest the inhibition of EPS and eDNA production may play an important role in the antibiofilm effect of Melo on PAO1 under the assay conditions used in this study.

### 3.4 Effect of Melo on the production of virulence factors and QS‐related genes in PAO1

**Figure 5:**
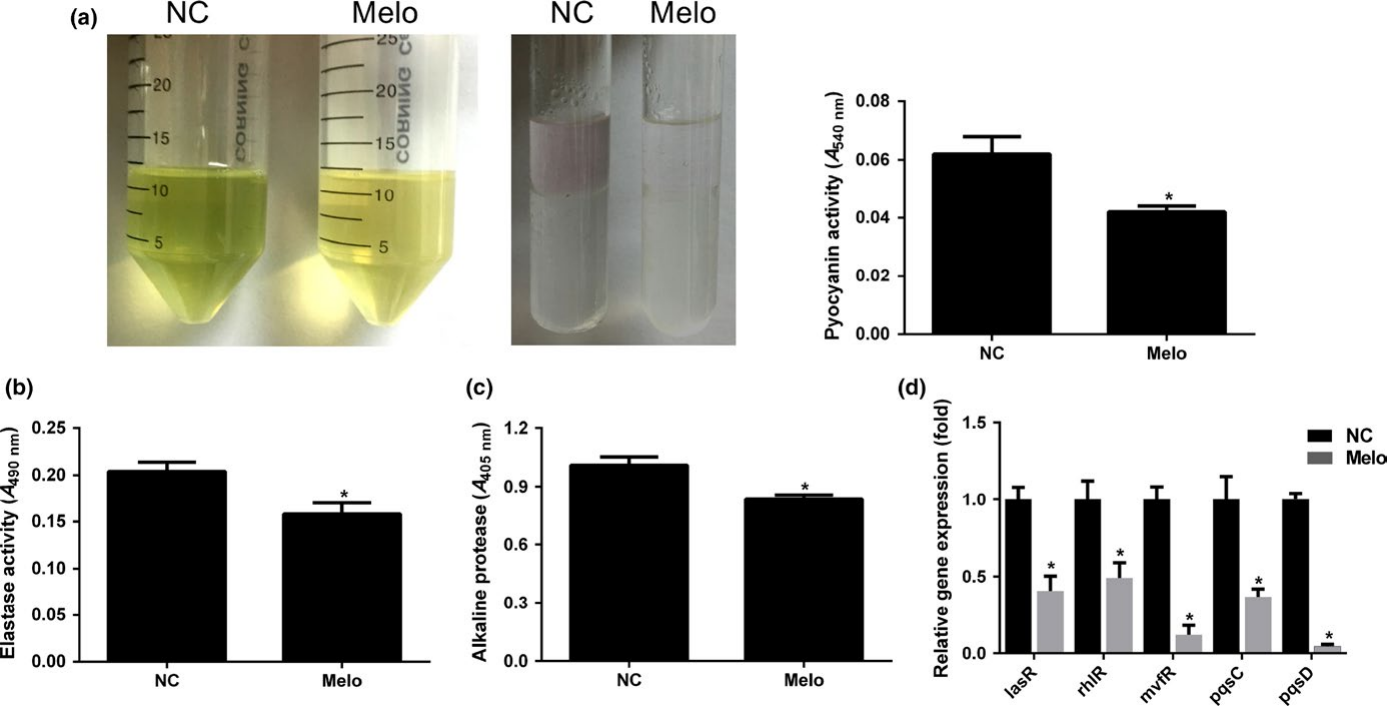
Effects of Melo on virulence factors and QS‐related genes. (a) Culture supernatant with (15.63 μg/mL) and without Melo was collected (left), and the pyocyanin was extracted by chloroform, and acidated with HCl (middle). Color change was measured by A540 nm. Elastase (b) and alkaline protease (c) were determined by measuring A490 nm and A405 nm, respectively. (d) Relative expression of lasR, rhlR, mvfR, and pqsC/D genes of PAO1 in the presence of 15.63 μg/mL Melo as determined by qPCR. * indicates that the mean of absorbance is statistically different from the control group with *p *<* *.05

Pyocyanin production of PAO1 was significantly inhibited by 15.63 μg/ml Melo compared to the group of negative control (Figure 5a right). After extraction by chloroform and acidification with HCl, the pigment of pyocyanin was turned to red from green (Figure 5a middle), then the absorbance was read at the wavelength of 540 nm (A540 nm), which indicated by 32% (*p* < .05) reduction in pyocyanin production (Fig. 5a left). Significant reduction (*p* < .05) in elastase (23%) (Figure 5b) and alkaline protease (16%) (Figure 5c) production in the presence of 15.63 μg/mL of Melo was observed. And qPCR showed that nearly 59%, 51%, 88%, 63%, and 95% reduction in the expression of *lasR*,* rhlR*,* mvfR*,* pqsC*, and *pqsD*, when compared to the control group, respectively, with 15.63 μg/mL of Melo (Figure 5e).

### 3.5 Susceptibility pattern of antimicrobials

TE, TOB, CTRX, OFLX, NFLX, CAZ, and DNase were selected to study the synergistic effects of Melo against biofilm formation of *P. aeruginosa*. MICs of 125 μg/ml for TE, 0.5 μg/ml for TOB, 8 μg/ml for CTRX, 8 μg/ml for OFLX, 4 μg/ml for NFLX, 0.5 μg/ml for CAZ, and >1,000 μg/ml for DNase were observed against *P. aeruginosa* standard strain PAO1.

### 3.6 Synergistic antibiofilm activity of Melo in combination with antibiotics

**Figure 6:**
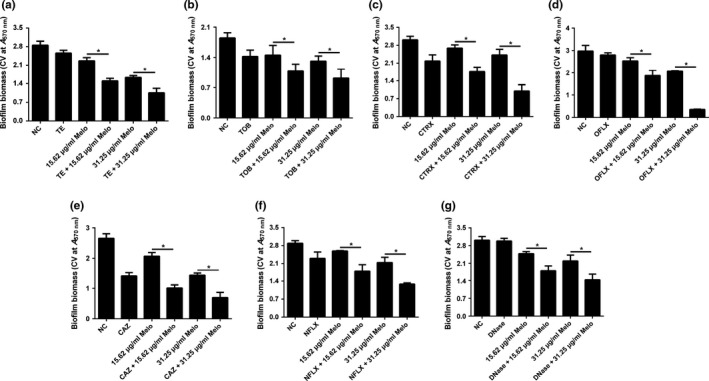
Synergistic effects of Melo combined with antimicrobials on PAO1 biofilm formation. Biofilms were exposed to Melo (15.63 or 31.25 μg/mL) alone or combined with TE, TOB, CTRX, OFLX, CAZ, NFLX, and DNase for 24 hr. Biofilms incubated with 1% DMSO was used as a negative control. And biofilm biomass was quantified by CV staining method. Bars indicated SE of the mean. * indicates that the mean of A570 nm is statistically different between groups with *p *<* *.05

At sub‐MIC levels (3.125 μg/ml for TE, 0.024 μg/ml for TOB, 3.125 μg/ml for CTRX and OFLX, 0.781 μg/ml for NFLX and CAZ, and 6.25 μg/ml for DNase), TOB (Figure 6b), CTRX (Figure 6c), NFLX (Figure 6f) and CAZ (Figure 6e) alone resulted in moderate reduction in biofilm formation for 24 hr incubation. However, TE (Figure 6a), OFLX (Figure 6d) and DNase (Figure 6g) did not affect the biofilm formation by PAO1 at the sub‐MIC levels. When these sub‐MIC antimicrobials were used in combination with Melo (15.63 or 31.25 μg/ml), a significant reduction of biofilm biomass (CV at A570 nm) was observed at 24 hr in comparison with the agents used alone, showing a synergistic effect (Figure 6).

## 4 DISCUSSION

Treatment of biofilm‐associated infections has become a challenge for clinicians owing to persistence of biofilms and rising multidrug resistance, antibiotics could be less active against bacteria embedded into a biofilm by which the antimicrobial activity of Melo should be studied not only against planktonic but also against biofilm‐associated organisms (del Prado et al., 2010).

In this work, we demonstrated that Melo could inhibit the biofilm formation of *P. aeruginosa* PAO1 and some of the clinical isolates at the concentration without limiting bacterial growth, probably by: (1) inhibiting of swimming, swarming, and twitching motility; (2) reducing the production of EPS and eDNA; (3) reducing the expression of QS‐related virulence factors and genes. Furthermore, we found that synergistic antimicrobial efficacy could be achieved when treating *P. aeruginosa* with Melo combined with antimicrobials. To our best knowledge, the antimicrobial effect of Melo and its potential synergistic effect when combined with antibiotics have not been explored. Therefore, this is the first work that highlights these effects.

Extracellular substances of biofilm are a complex mixture of biopolymers mainly including EPS, eDNA, and extracellular proteins. The major functions of these components comprise the primary adherence of bacterial cells to biotic or abiotic surface and protection against dehydration as well as environmental stress (Vu, Chen, Crawford, & Ivanova, 2009). Pel, Psl, and alginate are three vital biofilm EPS in *P. aeruginosa* (Sarabhai, Sharma, & Capalash, 2013; Vu et al., 2009). Pel is essential for the formation of pellicles at the air–liquid interface, while Psl is required for the initiate biofilm formation and biofilm structure maintenance (Vu et al., 2009). Alginate is regulated by gene operon of *alg44*, and is associated with chronic stages of biofilm‐mediated infection (Schurr, 2013). On the other hand, eDNA can be up to 50% more abundant than cellular DNA in biofilms of *P. aeruginosa* (Steinberger & Holden, 2005), in which it functions as an intercellular connector (Flemming & Wingender, 2010). eDNA plays a central role in biofilm formation by increasing cellular aggregation and surface adhesion, leading to increased biofilm strength and integrity (Das et al., 2015). Our result about the production of eDNA and expression of EPS‐related genes implies that the Melo treatment may inhibit biofilm formation by decreasing the eDNA, Psl, Pel, and alginate production.

There were several evidences that NASIDs have antipseudomonal effect (Alem & Douglas, 2004; Bandara, Sankaridurg, & Willcox, 2004; Kopolovic, Thraikill, Martin, Carey, & Cloutier, 1986; Naeem, Chadhury, Amjad, Rehaman, & Khan, 2012; Nicolau, Marangos, Nightingale, & Quintiliani, 1995; Stepanovic, Vukovic, Jesic, & Ranin, 2004; Umaru, Nwamba, & Kolo, 2009). For example, aspirin was reported to have a weak and broad‐spectrum antimicrobial activity towards some planktonic and biofilm cultures (Alem & Douglas, 2004; Nicolau et al., 1995; Stepanovic et al., 2004); diclofenac and ibuprofen could also limit the formation of biofilm by *Staphylococcus aureus* and *Escherichia coli* in the concentration obtained in the serum (Reslinski et al., 2015).

However, the mechanism of NASIDs impact on bacterial biofilm formation has not been fully explained. It is considered that this kind of effect should be due to inhibition of bacterial initial attachment (Demirag, Esen, Zivalioglu, Leblebicioglu, & Keceligil, 2007; Muller, Al‐Attar, Wolff, & Farber, 1998), reduction of EPS and protein syntheses (Farber & Wolff, 1992; Muller et al., 1998), and change of hydrophobicity (Naves et al., 2010). Another possible interpretation for biofilm inhibition is their impact on the QS system (Ulusoy & Bosgelmez‐Tinaz, 2013). QS system is a bacterial communication process that depends on the bacterial population density. *Pseudomonas aeruginosa* employs three QS signaling systems (LasR/LasI, RhlR/RhlI, and PQS) (Sauer, Camper, Ehrlich, Costerton, & Davies, 2002). The QS system of *P. aeruginosa* mainly possess two types of *N*‐acyl homoserine lactone (AHL) signaling system, the *lasR/I* system using 3‐oxo‐C12‐HSL and the *rhlR/I* system using C4‐HSL. Accumulation of those signaling molecules could trigger the corresponding receptors, and the AHL‐LasR/RhlR protein complexes not only positively regulate the AHL synthesis for itself, but they also act as activators for the promoter sites of the QS‐related operons (Naves et al., 2010) which control diverse array of functions like virulence, antibiotic resistance, swarming and motility, oxidative stress tolerance, and biofilm formation (Bhardwaj, Vinothkumar, & Rajpara, 2013). Recently, another type of QS system was revealed, namely PQS system. The synthesis of PQS depends on the *pqsABCDE* operon, which is positively controlled by the transcriptional regulator MvfR (Cao et al., 2001; Gallagher, McKnight, Kuznetsova, Pesci, & Manoil, 2002). Such QS system could regulate biofilm development, swarming motility, virulence, and pathogenicity mediated by the signal molecules of 2‐alkyl‐4(1*H*)‐quinolones (AQs) (Rampioni et al., 2010; Schertzer, Brown, & Whiteley, 2010).

Soheili et al. (2015) revealed that oxicams, both of piroxicam and Melo, could interact well with active sites of LasR and PqsE with the Ki of 119.43 nmol/L and 4.0 μmol/L by molecular docking and structural analysis methodology. LasR plays an important role in the initiation of the *P. aeruginosa* QS cascade system and PqsE can enhance this system. However, there is no in vitro or in vivo experimental evidence to support such speculation. Similarly, our study found that Melo showed significant inhibitory effect on *P. aeruginosa* biofilm formation in vitro, and Melo could significantly inhibit motilities, QS‐related virulence factors and genes expression. However, there was almost no effect of piroxicam on *P. aeruginosa* biofilm formation (data not shown).

With the increasing resistance of bacteria to traditional antibiotics, there is an urgent need for the alternatives to replace antibiotics for combating bacterial infections or adjunct therapies to be used in combination with antibiotics so that lower doses of the conventional antibiotics are required for treatment (Bhardwaj et al., 2013). These findings suggest that Melo could significantly inhibit the biofilm formation of *P. aeruginosa*, and the combination treatment of Melo with antimicrobials is a promising strategy to prevent biofilm infections. Despite the successful demonstration of synergy in vitro, it does not necessarily mean an improved clinical outcome, due to the complexity of the in vivo environment during infection. These results may open the door for the development of treatments to diminish biofilm‐related infection caused by *P. aeruginosa*.

## CONFLICT OF INTEREST

None declared.

## References

[mbo3545-bib-0001] Alem, M. A. , & Douglas, L. J. (2004). Effects of aspirin and other nonsteroidal anti‐inflammatory drugs on biofilms and planktonic cells of *Candida albicans* . Antimicrobial Agents and Chemotherapy, 48, 41–47. 14693516 10.1128/AAC.48.1.41-47.2004 PMC310207

[mbo3545-bib-0002] Balasubramanian, D. , Kong, K. F. , Jayawardena, S. R. , Leal, S. M. , Sautter, R. T. , & Mathee, K. (2011). Co‐regulation of beta‐lactam resistance, alginate production and quorum sensing in *Pseudomonas aeruginosa* . Journal of Medical Microbiology, 60, 147–156. 20965918 10.1099/jmm.0.021600-0 PMC3081088

[mbo3545-bib-0003] Bandara, B. M. , Sankaridurg, P. R. , & Willcox, M. D. (2004). Non‐steroidal anti inflammatory agents decrease bacterial colonisation of contact lenses and prevent adhesion to human corneal epithelial cells. Current Eye Research, 29, 245–251. 15590469 10.1080/02713680490516729

[mbo3545-bib-0004] Bhardwaj, A. K. , Vinothkumar, K. , & Rajpara, N. (2013). Bacterial quorum sensing inhibitors: Attractive alternatives for control of infectious pathogens showing multiple drug resistance. Recent Patents on Anti‐Infective Drug Discovery, 8, 68–83. 23394143 10.2174/1574891x11308010012

[mbo3545-bib-0005] Cao, H. , Krishnan, G. , Goumnerov, B. , Tsongalis, J. , Tompkins, R. , & Rahme, L. G. (2001). A quorum sensing‐associated virulence gene of Pseudomonas aeruginosa encodes a LysR‐like transcription regulator with a unique self‐regulatory mechanism. Proceedings of the National Academy of Sciences of the United States of America, 98, 14613–14618. 11724939 10.1073/pnas.251465298 PMC64730

[mbo3545-bib-0006] Chen, L. , & Wen, Y. M. (2011). The role of bacterial biofilm in persistent infections and control strategies. International Journal of Oral Science, 3, 66–73. 21485310 10.4248/IJOS11022 PMC3469879

[mbo3545-bib-0007] Chen, M. , Yu, Q. , & Sun, H. (2013). Novel strategies for the prevention and treatment of biofilm related infections. International Journal of Molecular Sciences, 14, 18488–18501. 24018891 10.3390/ijms140918488 PMC3794791

[mbo3545-bib-0008] CLSI . (2006). Clinical and Laboratory Standard Institute‐Methods for dilution antimicrobial susceptibility tests for bacteria that grow aerobically, approved standard. PA, USA: Wayne.

[mbo3545-bib-0009] Costerton, J. W. , Cheng, K. J. , Geesey, G. G. , Ladd, T. I. , Nickel, J. C. , Dasqupta, M. , & Marrie, T. J. (1987). Bacterial biofilms in nature and disease. Annual Review of Microbiology, 41, 435–464. 10.1146/annurev.mi.41.100187.002251 3318676

[mbo3545-bib-0010] Das, T. , Kutty, S. K. , Tavallaie, R. , Ibugo, A. I. , Panchompoo, J. , Sehar, S. , … Manefield, M. (2015). Phenazine virulence factor binding to extracellular DNA is important for *Pseudomonas aeruginosa* biofilm formation. Scientific Reports, 5, 8398. 25669133 10.1038/srep08398 PMC4323658

[mbo3545-bib-0011] Das, M. C. , Sandhu, P. , Gupta, P. , Rudrapaul, P. , De, U. C. , Tribedi, P. , … Bhattacharjee, S. (2016). Attenuation of *Pseudomonas aeruginosa* biofilm formation by Vitexin: A combinatorial study with azithromycin and gentamicin. Scientific Reports, 6, 23347. 27000525 10.1038/srep23347 PMC4802347

[mbo3545-bib-0012] del Prado, G. , Ruiz, V. , Naves, P. , Rodríquez‐Cerrato, V. , Soriano, F. , & del Carmen Ponte, M. (2010). Biofilm formation by *Streptococcus pneumoniae* strains and effects of human serum albumin, ibuprofen, N‐acetyl‐l‐cysteine, amoxicillin, erythromycin, and levofloxacin. Diagnostic Microbiology and Infectious Disease, 67, 311–318. 20638597 10.1016/j.diagmicrobio.2010.03.016

[mbo3545-bib-0013] Demirag, M. K. , Esen, S. , Zivalioglu, M. , Leblebicioglu, H. , & Keceligil, H. T. (2007). The effect of aspirin on adherence of slime‐producing, coagulase‐negative staphylococci to vascular grafts. Annals of Vascular Surgery, 21, 464–467. 17628264 10.1016/j.avsg.2006.06.006

[mbo3545-bib-0014] Dutta, N. K. , Mazumdar, K. , Baek, M. W. , Kim, D. J. , Na, Y. R. , Park, S. H. , … Park, J. H. (2008). In vitro efficacy of diclofenac against *Listeria monocytogenes* . European Journal of Clinical Microbiology and Infectious Diseases, 27, 315–319. 18188616 10.1007/s10096-007-0439-5

[mbo3545-bib-0015] Farber, B. F. , & Wolff, A. G. (1992). The use of nonsteroidal antiinflammatory drugs to prevent adherence of *Staphylococcus epidermidis* to medical polymers. Journal of Infectious Diseases, 166, 861–865. 1527423 10.1093/infdis/166.4.861

[mbo3545-bib-0016] Flemming, H. C. , & Wingender, J. (2010). The biofilm matrix. Nature Reviews Microbiology, 8, 623–633. 20676145 10.1038/nrmicro2415

[mbo3545-bib-0017] Gallagher, L. A. , McKnight, S. L. , Kuznetsova, M. S. , Pesci, E. C. , & Manoil, C. (2002). Functions required for extracellular quinolone signaling by *Pseudomonas aeruginosa* . Journal of Bacteriology, 184, 6472–6480. 12426334 10.1128/JB.184.23.6472-6480.2002 PMC135424

[mbo3545-bib-0018] Gnanadhas, D. P. , Elango, M. , Datey, A. , & Chakravortty, D. (2015). Chronic lung infection by *Pseudomonas aeruginosa* biofilm is cured by L‐Methionine in combination with antibiotic therapy. Scientific Reports, 5, 16043. 26521707 10.1038/srep16043 PMC4629202

[mbo3545-bib-0019] Høiby, N. , Bjarnsholt, T. , Moser, C. , Høiby, N. , Bjarnsholt, T. , Moser, C. , … Williams, C. (2015). ESCMID guideline for the diagnosis and treatment of biofilm infections 2014. Clinical Microbiology and Infection, 21, Suppl 1, S1–S25. 25596784 10.1016/j.cmi.2014.10.024

[mbo3545-bib-0020] Huang, R. , Li, M. , Ye, M. , Yang, K. , Xu, X. , & Gregory, R. L. (2014). Effects of Nicotine on *Streptococcus gordonii* Growth, Biofilm Formation, and Cell Aggregation. Applied and Environment Microbiology, 80, 7212–7218. 10.1128/AEM.02395-14 PMC4249166 25217021

[mbo3545-bib-0021] Kim, H. S. , & Park, H. D. (2013). Ginger extract inhibits biofilm formation by *Pseudomonas aeruginosa* PA14. PLoS ONE, 8, e76106. 24086697 10.1371/journal.pone.0076106 PMC3785436

[mbo3545-bib-0022] Kim, S. K. , Park, H. Y. , & Lee, J. H. (2015). Anthranilate deteriorates the structure of *Pseudomonas aeruginosa* biofilms and antagonizes the biofilm‐enhancing indole effect. Applied and Environment Microbiology, 81, 2328–2338. 10.1128/AEM.03551-14 PMC4357933 25616795

[mbo3545-bib-0023] Kopolovic, R. , Thraikill, K. M. , Martin, D. T. , Carey, L. C. , & Cloutier, C. T. (1986). A critical comparison of the hematologic, cardiovascular, and pulmonary response to steroids and nonsteroidal anti‐inflammatory drugs in a model of sepsis and adult respiratory distress syndrome. Surgery, 100, 679–690. 3764692

[mbo3545-bib-0024] Lidor, O. , AI‐Quntar, A. , Pesci, E.C. , & Steinberg, D . (2015). Mechanistic analysis of a synthetic inhibitor of the Pseudomonas aeruginosa LasI quorum‐sensing signal synthase. Scientific Reports,5, 16569. 26593271 10.1038/srep16569 PMC4655403

[mbo3545-bib-0025] Liu, H. , Zhao, Y. , Zhao, D. , Gong, T. , Wu, Y. , Han, H. , … Qu, D. (2015). Antibacterial and anti‐biofilm activities of thiazolidione derivatives against clinical staphylococcus strains. Emerging Microbes & Infections, 4, e1. 26038759 10.1038/emi.2015.1 PMC4317670

[mbo3545-bib-0026] Mah, T. F. , & O'Toole, G. A. (2001). Mechanisms of biofilm resistance to antimicrobial agents. Trends in Microbiology, 9, 34–39. 11166241 10.1016/s0966-842x(00)01913-2

[mbo3545-bib-0027] Muller, E. , Al‐Attar, J. , Wolff, A. G. , & Farber, B. F. (1998). Mechanism of salicylate‐mediated inhibition of biofilm in *Staphylococcus epidermidis* . Journal of Infectious Diseases, 177, 501–503. 9466548 10.1086/517386

[mbo3545-bib-0028] Naeem, M. , Chadhury, M. N. , Amjad, R. , Rehaman, S. , & Khan, K. (2012). Anti‐inflammatory, anti‐bacterial activity and structure‐activity relationships of substitutions on 4‐thiazolidinone derivatives ‐ Part‐1. Pakistan Journal of Pharmaceutical Sciences, 25, 731–739. 23009988

[mbo3545-bib-0029] Naves, P. , del Prado, G. , Huelves, L. , Rodríguez‐Cerrato, V. , Ruiz, V. , Ponte, M. C. , & Soriano, F. (2010). Effects of human serum albumin, ibuprofen and N‐acetyl‐L‐cysteine against biofilm formation by pathogenic *Escherichia coli* strains. Journal of Hospital Infection, 76, 165–170. 20615578 10.1016/j.jhin.2010.05.011

[mbo3545-bib-0030] Nesse, L. L. , Berg, K. , & Vestby, L. K. (2015). Effects of norspermidine and spermidine on biofilm formation by potentially pathogenic *Escherichia coli* and *Salmonella enterica* wild‐type strains. Applied and Environment Microbiology, 81, 2226–2232. 10.1128/AEM.03518-14 PMC4345383 25595767

[mbo3545-bib-0031] Nicolau, D. P. , Marangos, M. N. , Nightingale, C. H. , & Quintiliani, R. (1995). Influence of aspirin on development and treatment of experimental *Staphylococcus aureus* endocarditis. Antimicrobial Agents and Chemotherapy, 39, 1748–1751. 7486913 10.1128/aac.39.8.1748 PMC162820

[mbo3545-bib-0032] Projan, S. J. , & Youngman, P. J. (2002). Antimicrobials: New solutions badly needed. Current Opinion in Microbiology, 5, 463–465. 12354551 10.1016/s1369-5274(02)00364-8

[mbo3545-bib-0033] Qu, L. , She, P. , Wang, Y. , Liu, F. , Zhang, D. , Chen, L. , … Wu, Y. (2016). Effects of norspermidine on Pseudomonas aeruginosa biofilm formation and eradication. Microbiologyopen, 5, 402–412. 26817804 10.1002/mbo3.338 PMC4905993

[mbo3545-bib-0034] Rampioni, G. , Pustelny, C. , Fletcher, M. P. , Wright, V. J. , Bruce, M. , Rumbaugh, K. P. , … Williams, P. (2010). Transcriptomic analysis reveals a global alkyl‐quinolone‐independent regulatory role for PqsE in facilitating the environmental adaptation of *Pseudomonas aeruginosa* to plant and animal hosts. Environmental Microbiology, 12, 1659–1673. 20406282 10.1111/j.1462-2920.2010.02214.x PMC2901523

[mbo3545-bib-0035] Rashid, M. H. , & Kornberg, A. (2000). Inorganic polyphosphate is needed for swimming, swarming, and twitching motilities of *Pseudomonas aeruginosa* . Proceedings of the National Academy of Sciences of the United States of America, 97, 4885–4890. 10758151 10.1073/pnas.060030097 PMC18327

[mbo3545-bib-0036] Reslinski, A. , Dabrowiecki, S. , & Glowacka, K. (2015). The impact of diclofenac and ibuprofen on biofilm formation on the surface of polypropylene mesh. Hernia, 19, 179–185. 24366755 10.1007/s10029-013-1200-x PMC4372680

[mbo3545-bib-0037] Rybtke, M. , Hultqvist, L. D. , Givskov, M. , & Tolker‐Nielsen, T. (2015). *Pseudomonas aeruginosa* Biofilm Infections: Community Structure, Antimicrobial Tolerance and Immune Response. Journal of Molecular Biology, 427, 3628–3645. 26319792 10.1016/j.jmb.2015.08.016

[mbo3545-bib-0038] Sarabhai, S. , Sharma, P. , & Capalash, N. (2013). Ellagic acid derivatives from *Terminalia chebula* Retz. downregulate the expression of quorum sensing genes to attenuate *Pseudomonas aeruginosa* PAO1 virulence. PLoS ONE, 8, e53441. 23320085 10.1371/journal.pone.0053441 PMC3539995

[mbo3545-bib-0039] Sauer, K. , Camper, A. K. , Ehrlich, G. D. , Costerton, J. W. , & Davies, D. G. (2002). *Pseudomonas aeruginosa* displays multiple phenotypes during development as a biofilm. Journal of Bacteriology, 184, 1140–1154. 11807075 10.1128/jb.184.4.1140-1154.2002 PMC134825

[mbo3545-bib-0040] Schertzer, J. W. , Brown, S. A. , & Whiteley, M. (2010). Oxygen levels rapidly modulate *Pseudomonas aeruginosa* social behaviours via substrate limitation of PqsH. Molecular Microbiology, 77, 1527–1538. 20662781 10.1111/j.1365-2958.2010.07303.x PMC3098721

[mbo3545-bib-0041] Schurr, M. J. (2013). Which bacterial biofilm exopolysaccharide is preferred, Psl or alginate? Journal of Bacteriology, 195, 1623–1626. 23417492 10.1128/JB.00173-13 PMC3624548

[mbo3545-bib-0042] Soheili, V. , Bazzaz, B. S. , Abdollahpour, N. , & Hadizadeh, F. (2015). Investigation of *Pseudomonas aeruginosa* quorum‐sensing signaling system for identifying multiple inhibitors using molecular docking and structural analysis methodology. Microbial Pathogenesis, 89, 73–78. 26358567 10.1016/j.micpath.2015.08.017

[mbo3545-bib-0043] Steinberger, R. E. , & Holden, P. A. (2005). Extracellular DNA in single‐ and multiple‐species unsaturated biofilms. Applied and Environment Microbiology, 71, 5404–5410. 10.1128/AEM.71.9.5404-5410.2005 PMC1214645 16151131

[mbo3545-bib-0044] Stepanovic, S. , Vukovic, D. , Jesic, M. , & Ranin, L. (2004). Influence of acetylsalicylic acid (aspirin) on biofilm production by *Candida* species. Journal of Chemotherapy, 16, 134–138. 15216946 10.1179/joc.2004.16.2.134

[mbo3545-bib-0045] Stoodley, P. , Cargo, R. , Rupp, C. J. , Wilson, S. , & Klapper, I. (2002). Biofilm material properties as related to shear‐induced deformation and detachment phenomena. Journal of Industrial Microbiology and Biotechnology, 29, 361–367. 12483479 10.1038/sj.jim.7000282

[mbo3545-bib-0046] Ulusoy, S. , & Bosgelmez‐Tinaz, G. (2013). Nonsteroidal anti‐inflammatory drugs reduce the production of quorum sensing regulated virulence factors and swarm in motility in human pathogen *Pseudomonas aeruginosa* [corrected]. Drug. Research (Stuttg), 63, 409–413. 10.1055/s-0033-1343430 23599038

[mbo3545-bib-0047] Umaru, T. , Nwamba, C. O. , & Kolo, I. (2009). Antimicrobial activity of non‐steroidal anti‐inflammatory durgs with respect to immunological response: Diclofenac sodium as a case study. African Journal of Biotechnology, 8, 7332–7339.

[mbo3545-bib-0048] Vu, B. , Chen, M. , Crawford, R. J. , & Ivanova, E. P. (2009). Bacterial extracellular polysaccharides involved in biofilm formation. Molecules, 14, 2535–2554. 19633622 10.3390/molecules14072535 PMC6254922

[mbo3545-bib-0049] Walters, M. C. , Roe, F. , Bugnicourt, A. , Franklin, M. J. , & Stewart, P. S . (2003). Contributions of antibiotic penetration, oxygen limitation, and low metabolic activity to tolerance of Pseudomonas aeruginosa biofilms to ciprofloxacin and tobramycin. Antimicrobial Agents and Chemotherapy, 47, 317–323. 12499208 10.1128/AAC.47.1.317-323.2003 PMC148957

[mbo3545-bib-0050] Wang, W. H. , Wong, W. M. , Dailidiene, D. , Berg, D. E. , Gu, Q. , Lai, K. C. , … Wong, B. C. (2003). Aspirin inhibits the growth of *Helicobacter pylori* and enhances its susceptibility to antimicrobial agents. Gut, 52, 490–495. 12631656 10.1136/gut.52.4.490 PMC1773581

